# Interactions between tumor microenvironment and resistance to transarterial and systemic treatments for HCC

**DOI:** 10.20517/cdr.2024.212

**Published:** 2025-07-02

**Authors:** Maria Stella Franzè, Francesca Saffioti, Vasileios K. Mavroeidis

**Affiliations:** ^1^Department of Clinical and Experimental Medicine, University of Messina, Messina 98124, Italy.; ^2^Department of Gastroenterology and Hepatology, Oxford University Hospitals NHS Foundation Trust, John Radcliffe Hospital, Oxford OX3 9DU, UK.; ^3^Translational Gastroenterology and Liver Unit, John Radcliffe Hospital, Oxford OX3 9DU, UK.; ^4^University College London Institute for Liver and Digestive Health and Sheila Sherlock Liver Unit, Royal Free Hospital and University College London, London NW3 2QG, UK.; ^5^Departments of Transplant and General Surgery, North Bristol NHS Trust, Southmead Hospital, Bristol BS10 5NB, UK.; ^6^Department of HPB Surgery, Bristol Royal Infirmary, University Hospitals Bristol and Weston NHS Foundation Trust, Bristol BS2 8HW, UK.

**Keywords:** Hepatocellular carcinoma, extracellular matrix, transarterial chemoembolization, selective internal radiation therapy, systemic therapy, drug resistance, immune evasion, immune checkpoint inhibitors

## Abstract

Hepatocellular carcinoma (HCC) is a malignant tumor originating from hepatocytes, often developing against a backdrop of chronic inflammation and liver fibrosis. The primary risk factor for HCC is cirrhosis, and early detection is crucial for improving outcomes. Despite advances in treatment, the prognosis remains poor, with a 5-year survival rate of approximately 15%-38%. Growing evidence highlights the critical role of the tumor microenvironment (TME) in modulating tumor initiation, growth, progression, and, in some cases, suppression. The TME is a complex ecosystem composed of immune cells, cancer-associated fibroblasts, extracellular matrix components, and other factors such as growth factors and cytokines. By shaping tumor cell behavior, the TME facilitates immune evasion and contributes to resistance to treatment. Tumor-associated immune cells, including regulatory T cells, myeloid-derived suppressor cells, and tumor-associated macrophages, contribute to immune suppression and progression. On the other hand, immune activation via immune checkpoint inhibition has shown promise in improving outcomes, especially when combined with other treatments such as transarterial chemoembolization (TACE), selective internal radiation therapy (SIRT), and systemic therapies. Studies have demonstrated the potential of targeting the TME to enhance treatment efficacy, with immune modulation emerging as a key therapeutic strategy. This review explores the complex interactions within the TME in HCC, highlighting its role in therapy resistance and immune evasion. It also discusses current therapeutic approaches to target the TME to improve clinical outcomes in HCC patients.

## INTRODUCTION

Hepatocellular carcinoma (HCC) is the third leading cause of cancer-related deaths worldwide, with its incidence rising in Western countries^[1-3]^. Chronic liver inflammation triggers cycles of cell death and regeneration, which may culminate in cirrhosis and potentially progress to neoplastic transformation^[4]^. Indeed, cirrhosis is the predominant risk factor for HCC, carrying an annual risk of 2%-4% for progression to malignancy, and one-third of cirrhotic patients are estimated to develop HCC during their lifetime^[5,6]^. Therefore, early detection of HCC through surveillance is crucial in increasing the likelihood of patients accessing curative treatments^[7]^.

Despite advances in therapy, the prognosis for HCC remains poor. The 5-year survival rate is approximately 15%-38%, mainly due to late-stage diagnosis, therapeutic resistance, high recurrence rates, and frequent metastases^[8,9]^. For early-stage, liver-confined HCC, curative treatments such as liver transplantation (LT), surgical resection, and radiofrequency ablation are effective^[10,11]^. However, the majority of HCC cases (50%-60%) present at advanced stages, requiring systemic therapy^[12]^. Progress in immunology has revolutionized cancer treatment, positioning immuno-oncology as a promising approach to improve outcomes in HCC management^[13]^. However, challenges persist in accurately predicting and systematically addressing resistance mechanisms across diverse patient populations, often leading to suboptimal outcomes^[14]^. Tumor response to therapy is significantly shaped by the dynamic interplay between tumor cells and the surrounding tumor microenvironment (TME)^[15]^. Research has consistently shown that the TME is a highly intricate ecosystem that influences cancer progression, therapeutic efficacy, and resistance to treatment^[16,17]^.

This review aims to delve into the current understanding of the complex interplay within the TME in HCC, highlighting its role in modulating treatment responses, exploring its potential as a therapeutic target, and shedding light on how it drives immune resistance to both transarterial and systemic therapies.

## TME IN HCC

**Figure 1:**
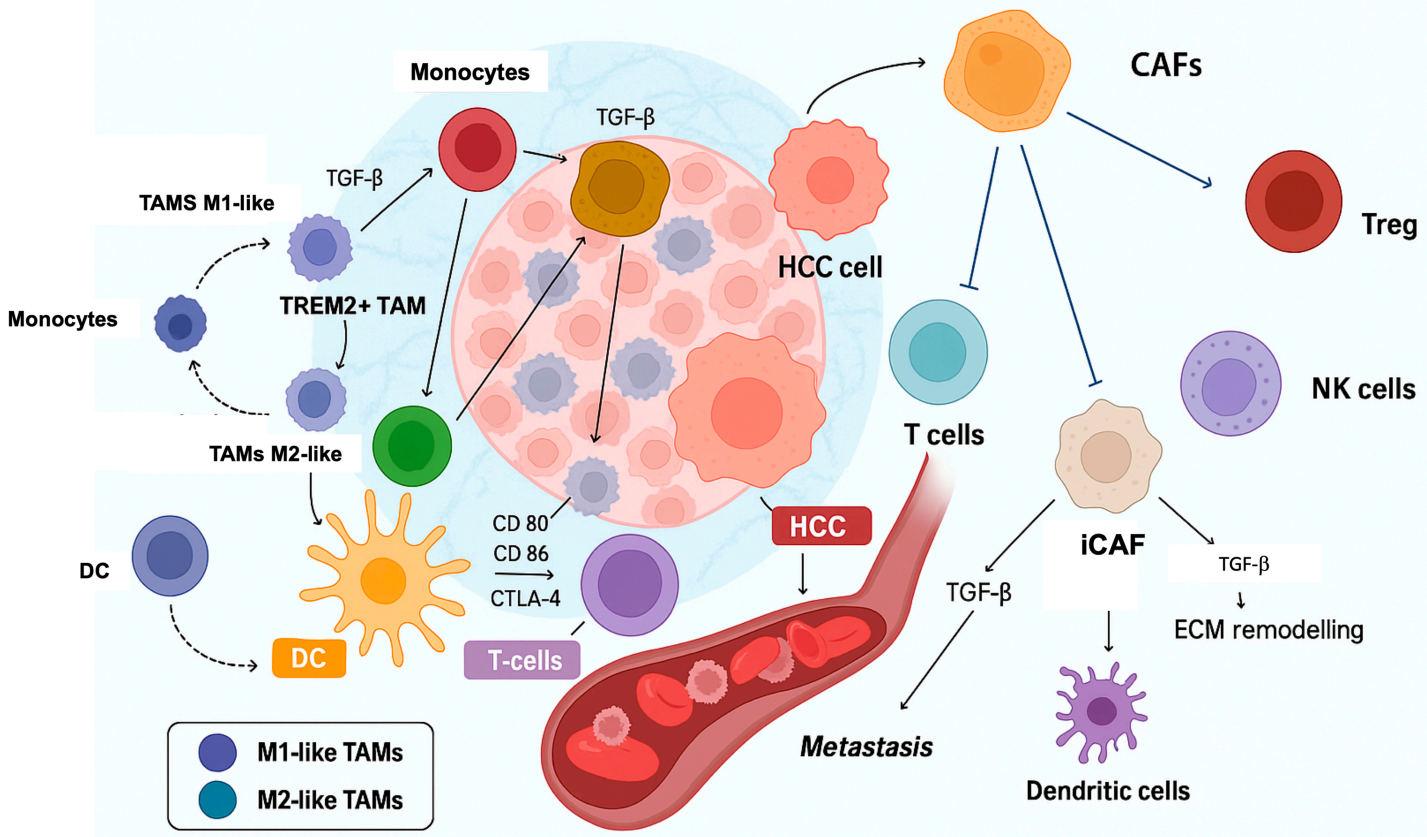
The TME balance in HCC (Created with Biorender using https://www.biorender.com/). CAFs: Cancer-associated fibroblasts; DC: dendritic cells; MDSC: myeloid-derived suppressor cells; NK: natural killer; TAMs: tumor-associated macrophages; Treg: regulatory T cells; TME: tumor microenvironment; HCC: hepatocellular carcinoma.

The TME in HCC, a complex and dynamic landscape, is a key player in tumor development, progression, and immune evasion^[18]^. It comprises cellular and non-cellular elements, including mesenchymal and immune cells, extracellular matrix (ECM), growth factors, pro-inflammatory cytokines, and bacterial products translocated via the enterohepatic circulation^[19]^. The cellular part includes impaired hepatocytes, liver progenitor cells, and a variety of immune cells. The other part encompasses the tumor stroma, enriched with growth and inhibitory factors, proteolytic enzymes, and cytokines with pro- and anti-inflammatory roles^[20]^ [Figure 1].

The nature and dynamics of the TME are shaped by the underlying causes of chronic liver disease and by genetic, epigenetic, and metabolic factors that influence cellular behavior. The immune landscape of the liver includes diverse populations such as neutrophils, monocytes, Kupffer cells, natural killer (NK) cells, natural killer T (NKT) cells, and circulating and resident lymphocytes (e.g., CD4+ and CD8+ T cells, B cells, and γδ T cells)^[21]^. In a healthy liver, these cells maintain homeostasis and regulate inflammation. Thus, the liver is predisposed to induce immune tolerance, a state of reduced immune responsiveness. Its unique architecture and blood flow dynamics facilitate this, particularly within the sinusoids, where immune cells interact with tolerogenic antigen-presenting cells, such as hepatocytes and Kupffer cells. Moreover, immune cells within the TME show contrasting roles because cytotoxic CD8+ T cells are pivotal for antitumor responses^[22]^.

In contrast, suppressive populations such as regulatory T cells (Tregs), myeloid-derived suppressor cells (MDSCs), tumor-infiltrating lymphocytes (TILs), and tumor-associated macrophages (TAMs) contribute to immune evasion and tumor progression^[23]^. Indeed, in chronic liver disease, prolonged necroinflammation disrupts the immune liver balance, fostering a microenvironment conducive to tumorigenesis. In this context, the TME supports tumor growth and provides a protective niche for cancer cells, enabling immune escape^[18]^.

Stromal cells such as cancer-associated fibroblasts (CAFs) are key elements that remodel the ECM and secrete factors that enhance tumor invasion and metastasis. Their activation is driven by signals such as transforming growth factor-β (TGF-β) and platelet-derived growth factor (PDGF)^[24]^. Moreover, the ECM plays a crucial role in modulating tissue stiffness while serving as a reservoir for signaling molecules such as vascular endothelial growth factor (VEGF) and hypoxia-inducible factor-1a (HIF-1a), which are upregulated under hypoxic conditions commonly associated with rapidly growing tumors. This promotes angiogenesis, further sustaining tumor survival^[14]^. Extracellular vesicles (EVs), including exosomes, facilitate intercellular communication within the TME, playing critical roles in immune modulation, angiogenesis, and metastasis^[25]^. In addition to stromal and immune cells, cancer stem cells (CSCs) represent a subset within the TME, characterized by their ability to self-renew and differentiate. These cells contribute significantly to tumor heterogeneity, resistance to therapy, and recurrence. Crosstalk between CSCs, cancer cells, and stromal components drives aberrant signaling pathways, such as Wnt, which promote tumor growth, invasion, and metastasis^[26]^.

Moreover, the gut microbiota significantly impacts the TME through the gut-liver axis, where dysbiosis and microbial translocation amplify inflammatory signaling and fibrosis, thereby accelerating HCC pathogenesis^[27]^. Understanding the intricate interactions within the HCC microenvironment, including the roles of stromal components, immune cells, CSCs, and microbial influences, opens avenues for therapeutic innovation. Targeting these elements, as this research suggests, has the potential to significantly improve treatment outcomes for HCC patients^[28]^.

Furthermore, recent research utilizing single-cell RNA sequencing (scRNA-seq) has revealed significant variability among HCC cells between different patients, while the TME shows more consistent gene expression patterns across individuals^[29]^. Using RNA and clinical data from 371 patients in The Cancer Genome Atlas (TCGA), a 3-gene signature was developed and validated to classify patients into high- and low-risk groups, with low-risk patients showing significantly better survival outcomes. Functional enrichment analyses highlighted ECM architecture, tumor-associated pathways, and immune microenvironment suppression in high-risk groups. DLAT, a key gene in the signature, was shown to promote liver cancer cell migration, proliferation, and drug resistance, making it a potential prognostic marker and therapeutic target^[30]^. These findings suggest that therapies targeting the TME, particularly immunotherapies, hold remarkable promise for treating patients with HCC, inspiring and motivating further research and development in this area^[31]^.

### Immune activation

The processes of tumor cell proliferation, necrosis, and specific therapeutic interventions continuously lead to the release of cancer cell antigens. These antigens, crucial for the immune response, are captured by dendritic cells (DCs) via toll-like receptors (TLR2 and TLR4). The captured antigens trigger the maturation of DCs, enabling their migration to lymph nodes^[32,33]^. This migration is guided with precision by chemokines, underscoring the meticulous orchestration of the immune response. In the lymph nodes, DCs present the antigens to CD8+ cytotoxic T lymphocytes (CTLs) via co-stimulatory molecules like CD40. The activation of CTLs is further facilitated by IFN-γ from NK cells and TH1 cells, tumor necrosis factor-alpha (TNF-α), and IL-12 from macrophages, and chemokines (CXCL-9, CXCL-10, CCL-5)^[34]^. The interactions between lymphocyte function-associated antigen 1 (LFA-1) and intercellular adhesion molecule-1 (ICAM-1) enable CTLs to infiltrate tumors, recognize cancer cells via T cell receptors, and induce cell death^[34]^.

In recent years, high-resolution techniques such as scRNA-seq and spatial transcriptomics have expanded our understanding of T cell heterogeneity and function within the TME^[29]^. Single-cell analyses in HCC have revealed that CD8+ T cells exhibited a spectrum of exhaustion, marked by distinct transcriptional profiles that reflect varying degrees of dysfunction and differing capacities to respond to immune checkpoint (IC) inhibition^[35]^. A unique subset of CXCL13+ CD8+ T cells has been identified within HCC tissues, notably enriched in individuals showing favorable responses to anti-PD-1 therapy. These cells appear to play a pivotal role in establishing immune cell niches and coordinating antitumor responses. Additionally, a population of stem-like CD8+ T cells characterized by TCF1 expression persists in HCC and serves as a self-renewing source of effector T cells during PD-1 blockade, highlighting their relevance as therapeutic targets and biomarkers. These cells have emerged as important targets and biomarkers for immunotherapy^[36]^.

However, the immune system faces a significant challenge in the form of immune checkpoints (ICs), such as CTLA-4 and PD-1 on T cells. These ICs interact with CD80/86 and PD-L1 on DCs, effectively suppressing the immune response. The upregulation of PD-1 on T cells and PD-L1 on DCs by immunosuppressive cytokines (e.g., IL-10, TGF-β, prostaglandin E2) further exacerbates this challenge^[37]^. Additionally, VEGF produced by cancer cells suppresses T cell infiltration by activating the PI3K/AKT pathway, adding another layer of complexity to the immune response^[38]^.

### Immune suppression

Several immune cells are involved in the immune suppression mechanisms occurring in the TME of HCC^[39]^. Neutrophils, integral to immunity, play dual roles in tumor development. Initially, tumor-associated neutrophils (TANs) display an anti-tumorigenic phenotype (N1), but under the influence of factors like TGF-β and CXCL6, CAFs polarize TANs toward a pro-tumorigenic (N2) phenotype^[40]^. N2 TANs form neutrophil extracellular traps (NETs) via NETosis, enhancing tumor growth, invasiveness, and resistance to therapies by activating TLR4/9-COX2 pathways. N2 TANs also contribute to immunosuppression by inhibiting neutrophil recruitment through the PD-1/PD-L1 pathway^[41-44]^. TANs recruit macrophages and Tregs via CCL-2 and CCL-17, promoting resistance to treatments like sorafenib and releasing pro-metastatic factors, which stimulate angiogenesis and enhance tumor cell migration^[45]^.

Beyond CD8+ T cells, regulatory CD4+ T cells (Tregs) are recognized as key mediators of immunosuppression in the TME. Surface molecules such as CCR8 and TIGIT have been proposed as selective targets to deplete intratumoral Tregs while sparing peripheral immune regulation^[46]^.

DCs are specialized immune cells that capture tumor antigens and present them to naïve T cells, initiating immune activation and their differentiation into effector T cells. DCs are categorized based on their differentiation stage and environment into conventional DCs (cDCs), plasmacytoid DCs (pDCs), and inflammatory DCs^[47]^. In HCC, reduced levels of cDCs and pDCs and impaired co-stimulatory molecule expression create a favorable TME for cancer progression. Specifically, BDCA2+ pDCs recruit Treg cells that secrete IL-10, promoting immunosuppression. DC subsets, such as LAMP3+ DCs, are linked to T cell exhaustion and immunosuppressive regulation^[48]^. The TME further disrupts dendropoiesis, polarizing DCs toward immunosuppressive phenotypes. DC-based vaccines and immunotherapies are under clinical evaluation and show promise in enhancing antitumor immunity and improving T cell responses^[49]^.

The liver also has a high macrophage density, and TAMs play dual roles depending on their phenotype: pro-inflammatory M1 macrophages (CD86+) or immunosuppressive M2 macrophages (CD163+, CD206+)^[50]^. TAMs, marked by CD68+, are common in HCC and associated with aggressive disease phenotypes. Elevated levels of M2 macrophages relative to M1 macrophages correlate with metastasis, immunosuppression, angiogenesis, and drug resistance^[51]^. At the same time, TAMs promote tumor progression by secreting cytokines such as IL-6, IL-10, TNF-α, and VEGF, which inhibit T cells and NK cells and promote Treg differentiation^[52,53]^.

Monocytes recruited by CCL-2 initially have antitumor functions but later promote tumor progression. In advanced HCC, CD14+ monocytes expressing PD-L1, IL-10, and CCL-1 suppress immune responses, aiding immune escape and angiogenesis. MDSCs, immature immune cells, are classified into polymorphonuclear (PMN) and monocytic (M-MDSCs)^[54]^. PMN-MDSCs suppress immunity, while M-MDSCs utilize IL-10, TGF-β, nitric oxide, and PD-L1 to inhibit NK cell activity and promote Tregs. These cells suppress excessive immune responses and inflammation and are classified into natural Tregs (FOXP3+, CD25+, CTLA-4+) and induced Tregs (FOXP3+, CD4+). They inhibit T cell activation and function through genetic and cytokine-mediated mechanisms, and their infiltration into the TME suppresses immunity. However, CCR4+ Tregs are implicated in resistance to immune checkpoint inhibitors (ICIs) in HCC patients^[55]^.

Indeed, CAFs are specialized fibroblasts originating from mesenchymal cells, cancer cells, or bone marrow stem cells. They are classified into myofibroblastic CAFs (myCAFs) and inflammatory CAFs (iCAFs)^[56]^. myCAFs secrete ECM components and respond to TGF-β, while iCAFs produce high levels of IL-6 and IL-11. CAFs contribute to tumor progression through angiogenesis by releasing VEGF and CXCL-12, invasion, metastasis, immune suppression by recruiting immunosuppressive cells, and therapy resistance through recruitment of cytokines^[57]^.

### Detection and monitoring of the TME in HCC

Characterizing the TME in HCC is essential for understanding disease progression and optimizing therapeutic strategies. Traditional histopathological methods provide limited insights into the complexity of the TME^[58]^. Recent advances have led to the development of high-resolution, multiparametric technologies capable of profiling both spatial and functional aspects of the TME.

Among the most widely used methods, scRNA-seq and spatial transcriptomics enable the deconvolution of heterogeneous cell populations and the identification of cellular subtypes and states within the TME^[59]^. These technologies reveal the transcriptional activity of individual cells and preserve spatial context, which is critical for mapping cell-cell interactions and immune niches. Multiplex immunohistochemistry (mIHC) and imaging mass cytometry (IMC) enable simultaneous visualization of dozens of proteins in tissue sections, supporting the phenotypic and functional characterization of immune and stromal cells *in situ*^[60,61]^.

Flow cytometry and mass cytometry (CyTOF) remain invaluable tools for profiling immune populations in both tumor tissues and peripheral blood, offering dynamic quantification of immune signatures over time. Looking toward future applications, liquid biopsy technologies - such as the analysis of circulating tumor DNA (ctDNA), circulating tumor cells (CTCs), EVs, and tumor-derived exosomes - are gaining momentum as minimally invasive approaches to monitor changes in the TME^[62]^. These tools allow for longitudinal sampling, providing insights into tumor evolution, immune escape, and therapy-induced shifts in the microenvironment. Moreover, advanced molecular imaging techniques, including positron emission tomography (PET) with immune-specific tracers and contrast-enhanced MRI targeting stromal components, may offer non-invasive, real-time visualization of TME components. Together, these approaches promise to transform our ability to monitor the TME dynamically, enabling personalized treatment adjustments and early identification of therapeutic resistance.

## TME IN HCC PATIENTS TREATED WITH TRANSARTERIAL CHEMOEMBOLIZATION

According to the Barcelona Clinic Liver Cancer (BCLC) 2022 update and the last European Association for the Study of the Liver (EASL) clinical practice guidelines on the management of HCC, transarterial chemoembolization (TACE) is recommended as the first-line treatment for intermediate-stage HCC^[11,63]^. However, TACE is also commonly employed in early-stage HCC as a bridging for LT or in advanced stages for downstaging to curative therapies such as liver resection or in combination with systemic therapies^[10]^. The procedure works by directly delivering chemotherapy agents to the tumor while simultaneously obstructing its blood supply. It achieves tumor regression in up to 50% of patients by combining the direct cytotoxic effects of intra-arterial chemotherapy delivery with ischemic damage resulting from the interruption of blood flow^[64]^. Two main drug delivery methods exist for TACE: conventional TACE (cTACE) and the drug-eluting bead TACE (DEB-TACE). The principle behind cTACE is the injection into tumor-feeding arteries of a viscous emulsion that combines a chemotherapeutic agent (e.g., doxorubicin, idarubicin, or cisplatin) with iodized oil (Lipiodol®) and is often followed by embolization with gelatin sponges or other agents to block blood flow^[65]^. This mixture can persist in the tumor tissue for over a year due to the tumor’s siphon effect on iodized oil. Repeated chemotherapy increases toxicity, while arterial embolization intensifies tumor ischemia and hypoxia. These conditions reshape the TME, enabling immune evasion and promoting glycolysis, which accelerates tumor growth, recurrence, and metastasis^[66]^. In DEB-TACE, the chemotherapeutic agent (e.g., doxorubicin, pirarubicin, epirubicin, and Adriamycin) is incorporated into microspheres for slow drug release. However, the microspheres cannot penetrate the peribiliary capillary plexus and reach the tumor directly, and the drug is released in the distal arterioles and spreads via diffusion, convection, or both^[67]^. The immunosuppressive nature of the hepatic microenvironment significantly limits the efficacy of chemotherapeutic agents. Moreover, these agents further modify the composition of hepatic inflammatory cell populations, complicating their therapeutic impact. Doxorubicin, the most widely used drug in TACE, directly induces apoptosis, triggering immunogenic cell death and immune activation in the liver. Following TACE, the peripheral immune profile can show an increased CD4/CD8 ratio, a rise in Th17 cells, and a significant reduction in T regs. Additionally, TACE can stimulate the early release of pro-inflammatory cytokines, such as IL-22 and IL-6^[68]^. Apart from doxorubicin, among the other chemotherapeutic agents, idarubicin stands out due to its higher lipophilicity and smaller polar molecular surface area^[69]^. This enables greater idarubicin concentration in Lipiodol®, leading to improved permeability across biological membranes, and increased contact time between the drug and cancer cells, potentially improving therapeutic outcomes. Idarubicin’s superior cytotoxicity is particularly evident in chemo-resistant HCC cells, strongly suggesting a therapeutic advantage over doxorubicin, and a promising alternative.

The therapeutic effect of TACE differs between individuals, giving rise to the concept of TACE refractoriness or failure^[70-72]^. The mechanisms underlying TACE failure remain unclear, though key contributors are thought of as resistance to chemotherapeutic agents following repeated TACE treatments. Key contributors include VEGF upregulation-driven angiogenesis and neovascularization^[73]^. The effectiveness of TACE is partially attributed to its ability to modulate innate and adaptive immunity^[74,75]^. Following intra-arterial chemotherapy delivery and tumor embolization, the release of cellular debris, pro-inflammatory cytokines, and danger-associated molecular patterns (DAMPs) has a priming effect on adaptive immunity^[76,77]^. However, the precise functional characteristics of the TME in response to TACE are unknown.

Spontaneous TACE-induced T cell responses have been associated with improved clinical outcomes, suggesting that the modulation of adaptive immunity may play a key role in the effectiveness of TACE. A study evaluated the impact of TACE on T cell function by analyzing intratumoral (IT), peritumoral (PT), and non-tumoral (NT) tissues in 119 patients resected or transplanted with or without prior TACE treatment^[78]^. Fifty-eight TACE-treated patients had liver samples displaying reduced IT Tregs CD4+/FOXP3+ (*P* = 0.006) and immune-exhausted T cells CD8+/PD-1+ (*P* < 0.001) compared to 61 who did not receive TACE before liver surgery, which correlated with improved recurrence-free survival (*P* = 0.005). Transcriptomic analysis revealed upregulation of pro-inflammatory pathways, including IRF2 (*P* = 0.01), a factor in immune evasion^[78]^. These findings highlight the dual effects of TACE on the TME and support the integration of immunotherapy with TACE to enhance treatment outcomes. The landscape of post-TACE HCC was also investigated with scRNA-seq. This method and functional assays revealed a reduction in CD8+ T cells and an increase in TREM2+ TAMs in the post-TACE TME. TREM2+ TAMs suppress CD8+ T cells by altering CXCL9 and Galectin-1 secretion, with Galectin-1 promoting PD-L1 overexpression in endothelial cells. TREM2 deficiency enhanced CD8+ T cell infiltration, inhibited tumor growth, and improved the efficacy of anti-PD-L1 therapy. These findings highlight TREM2 as a potential immunotherapeutic target to counter HCC progression and improve post-TACE outcomes^[79]^.

Several scoring systems have been developed to predict outcomes following TACE, utilizing routinely measured biomarkers generally specific to HCC rather than the TACE procedure^[80,81]^. Thus, using associations between transcriptomic data and the response to TACE, a gene expression database from Gene Expression Omnibus (GEO) was employed to develop a predictive gene signature for the response to TACE. At the same time, an external validation confirmed the model’s ability to distinguish survival outcomes in patients receiving adjuvant or post-recurrence TACE^[82,83]^. Furthermore, the trans-arterial procedure can stimulate the production of proangiogenic cytokines and trigger immunogenic cell death, leading to tumor angiogenesis and alterations in the tumor immune cell microenvironment^[84]^. TACE refractoriness may lead to a poor prognosis in patients with HCC^[70,72]^. Consequently, identifying novel biomarkers to predict TACE refractoriness and prognosis has become critical in advancing HCC treatment strategies. In this context, TACE refractoriness diagnostic (TRD) and prognostic (TRP) scores were developed with four novel TACE refractoriness-related genes (TRGs). Tumors with a high TRP score exhibited an immunosuppressive phenotype, with increased infiltration of Tregs and macrophages, defining a good response to ICIs and sorafenib^[85]^.

The biomarker NABP1 was validated in a cohort of patients with HBV-related HCC^[86]^. In particular, scRNA-seq and analysis of HBV-related HCC specimens revealed that TACE treatment increases tumor heterogeneity and induces a pro-inflammatory microenvironment. Bulk data analysis linked a higher proportion of NABP1+ hepatocytes with poor TACE response and prognosis, suggesting NABP1 as a valuable tool for identifying patients likely to respond to first-line TACE and shed light on the mechanisms of postoperative progression^[86]^. Another study investigated the role of MDSCs, particularly M-MDSCs and early-stage MDSCs (eMDSCs), in HCC and their changes following microparticle TACE (mTACE)^[87]^. Among 75 HCC patients, 16 with liver cirrhosis, and 20 healthy controls, M-MDSC levels were significantly elevated in HCC and correlated with aggressive clinical features such as tumor size, vascular invasion, and metastasis. Post-mTACE, M-MDSC levels significantly decreased, while eMDSC levels remained unchanged. These findings underscore M-MDSCs as key players in HCC progression and suggest mTACE’s potential to mitigate immunosuppression, paving the way for combined immunotherapeutic approaches in HCC treatment^[87]^.

In another context, biomarkers for predicting TACE response in HCC patients were explored and correlated with the TME and pre-TACE radiomics features^[88]^. Five key genes (ADH1C, CXCL11, EMCN, SPARCL1, LIN28B) were incorporated into a TACE Failure Signature (TFS) model, effectively predicting TACE response and overall survival (OS). A radiomics-based Rad-score model was developed using imaging data, showing significant correlations with TFS scores and hub gene expression. High TFS scores were linked to an immunosuppressive TME and poor responses to immunotherapy (PD-1, CTLA-4 inhibitors)^[88]^. This integrated biomarker approach demonstrated insights into TACE efficacy, tumor immune dynamics, and potential personalized treatment strategies.

The observed changes in inflammatory populations and inhibitory ICIs following TACE highlight the need for further investigation into combining TACE with ICIs. Indeed, TACE induces liver tumor cell necrosis, but also activates T cell responses, while simultaneously increasing PD-1/PD-L1 expression in the TME^[89]^. This makes anti-PD-1 drugs potential candidates for enhancing therapeutic outcomes. Combining TACE with ICIs can reduce the accumulation of MDSCs and Tregs, potentially improving immune response^[90]^. However, this combination is under investigation in clinical trials.

## TME IN HCC PATIENTS TREATED WITH SELECTIVE INTERNAL RADIATION THERAPY

Selective internal radiation therapy (SIRT) is a recent therapeutic alternative option proposed for the management of intermediate-stage HCC (BCLC stage B) in patients who are not eligible for other locoregional therapies, as well as for locally advanced HCC (BCLC stage C)^[91,92]^. SIRT has demonstrated efficacy in tumor downstaging and delaying disease progression with sustained therapeutic response despite the Y90 isotope’s relatively short half-life. Maximal clinical effects, such as tumor regression and reduced serum alpha-fetoprotein levels, emerge 3-6 months after treatment. However, the mechanisms behind this delayed yet sustained antitumor response remain unclear^[93]^. The feasibility and safety of this treatment were initially demonstrated in numerous retrospective multicenter studies and later validated in prospective studies. The widespread use of SIRT in recent years has led to the study of prognostic factors that could predict a response to this treatment^[94]^. This search is necessary in the context of SIRT to better select HCC patients who may benefit from this treatment. In this context, the TME may play a significant role. Ionizing radiation from therapies like TARE can provoke modest inflammation in the TME, leading to upregulation of chemokines and cytokines, as demonstrated in preclinical models^[95,96]^. Yet, a comprehensive understanding of how these changes affect local and systemic immunity in cancer patients is lacking. To address this, researchers analyzed immune profiles of surgically resected HCC tumors post-Y90-RE using time-of-flight mass cytometry (CyTOF) for high-dimensional immunophenotyping. This revealed significant antitumor immune responses, including activating immune subsets and recruiting granzyme B-positive CD8+ T cells^[97]^. Complementary next-generation sequencing (NGS) identified pathways facilitating these immune cell infiltrations. A recent Asian study investigated the immunological effects of Yttrium-90 radioembolization (Y90-RE) in HCC, demonstrating its ability to activate both local and systemic immune responses, contributing to sustained therapeutic outcomes. Key findings include increased infiltration of immune cells (CD8+ T cells, NK cells, and NKT cells) in treated tumors and upregulation of innate and adaptive immunity genes. Chemotactic pathways involving CCL5 and CXCL16 were correlated with the recruitment of GB+ CD8+ T cells activated in tumors treated with SIRT. Comparing peripheral blood mononuclear cells before and after SIRT, the authors observed an increase in TNF-α on CD8+ and CD4+ T cells, as well as an increase in the percentage of antigen-presenting cells after SIRT^[98]^.

Moreover, a prediction model based on pretreatment PBMC immune profiles has been developed to identify sustained responders, offering the potential for personalized therapeutic approaches. These data strongly suggest that the characteristics of the intratumoral immune microenvironment before treatment play a significant role in the sensitivity of HCC to SIRT. As predictive factors for response to SIRT are still poorly defined, determining the pretreatment immune profile could help better select patients and provide a rationale for defining new therapeutic strategies, such as the combination of SIRT and immunotherapy^[99]^. This research underscores the potential of the intratumoral immune microenvironment in the sensitivity of HCC to SIRT, sparking further interest in this area of study.

Table 1 summarizes studies investigating TME in HCC and factors related to response to TACE and SIRT.

**Table 1:** Studies investigating TME in HCC and factors related to response to TACE and SIRT

**Author (year)**	**Country**	**Key findings**
Huang *et al.*^[84]^ (2016)	China	TACE-induced hypoxia in HCC elevates HIF-1α and COX-2 expression, promoting EMT and increasing tumor invasion and metastasis. These changes may underlie poor prognosis in HCC patients post-TACE treatment
Tampaki *et al.*^[75]^ (2020)	Greece	Serum TIM-3 levels are elevated in advanced HCC and increase significantly after TACE, with higher post-TACE levels in complete responders, suggesting a potential role in antitumor immunity
Pinato *et al.*^[78]^ (2021)	Italy/Japan/Spain/UK	TACE reduces intratumoral densities of immune-exhausted cytotoxic T cells and Tregs while upregulating pro-inflammatory pathways, such as IRF2 expression, in the TME, supporting the potential for combining immunotherapy with TACE to enhance antitumor immunity
He *et al.*^[85]^ (2022)	China	A novel TRP score based on four genes (TTK, EPO, SLC7A11, PON1) accurately predicts TACE refractoriness and prognosis in HCC, identifying tumors with immunosuppressive phenotypes and guiding therapy decisions, including ICIs and sorafenib
Tang *et al.*^[82]^ (2022)	China	A 10-gene expression model accurately predicts HCC patient response to TACE, linking nonresponse to higher tumor stemness and distinct immune profiles, such as increased M0 macrophages
Tan *et al.*^[79]^ (2023)	China	TACE promotes TREM2+ TAMs, which suppress CD8+ T cells and contribute to HCC progression. TREM2 deficiency enhances CD8+ T cell activity and improves anti-PD-L1 therapy, making TREM2 a potential immunotherapy target post-TACE
Wang *et al.*^[86]^ (2024)	China	TACE induces tumor heterogeneity and a pro-inflammatory microenvironment, with elevated interactions between NABP1+ malignant hepatocytes, neutrophils, and CD8+ T cells linked to poor prognosis. NABP1 emerges as a potential biomarker for identifying HCC patients likely to respond to TACE
Yue *et al.*^[87]^ (2024)	China	mTACE effectively reduces immunosuppressive mMDSC levels in HCC patients, correlating with improved modulation of the TME. These findings position mMDSCs as potential therapeutic targets and support combining mTACE with immunotherapy for enhanced HCC management
Wang *et al.*^[88]^ (2024)	China	A transcriptomic biomarker (TFS) predicts TACE response in HCC patients, correlating with radiomics features, an immunosuppressive TME, and poor immunotherapy outcomes, offering a tool for personalized treatment strategies
Chew *et al.*^[98]^ (2019)	Singapore	Y90-radioembolization activates local and systemic immune responses, including increased granzyme B+CD8+T cells and antigen-presenting cells, correlating with sustained clinical response. A prediction model using pretreatment immune profiles identifies potential responders to Y90-RE

TME: Tumor microenvironment; HCC: hepatocellular carcinoma; TACE: transarterial chemoembolization; SIRT: selective internal radiation therapy; HIF-1α: hypoxia-inducible factor-1α; EMT: epithelial-to-mesenchymal transition; TIM-3: T cell immunoglobulin and mucin domain 3; IRF2: interferon regulatory factor 2; TRP: TACE refractoriness prognostic score; ICIs: immune checkpoint inhibitors; TREM2: triggering receptor expressed on myeloid cells 2; TAMs: tumor-associated macrophages.

## TME IN HCC PATIENTS TREATED WITH SYSTEMIC THERAPIES

According to international guidelines, systemic therapy is the preferred treatment for patients with advanced HCC (BCLC stage C) and those in stages A or B when other therapies are not feasible or have failed^[11,63]^. Immunotherapy uses the body’s natural defense mechanisms to fight tumor cells. ICs are molecules expressed on lymphocytes that regulate T cell functions and play a key role in tumor-associated immune tolerance. PD-L1 and PD-L2, for example, are expressed by various immune cells - including T lymphocytes, NK cells, and myeloid cells - as well as by tumor cells. Their interaction with PD-1 receptors on effector T cells suppresses immune activity, contributing to an immunosuppressive TME^[26]^. ICIs work by blocking these pathways, thereby restoring and enhancing antitumor immune responses with the TME^[100]^.

In fact, the TME plays a pivotal role in modulating immune responses, particularly those involving CD8+ CTLs, that infiltrate both the tumor core and peritumoral regions, where their presence correlates with improved clinical outcomes^[101]^. However, the TME exerts strong immunosuppressive pressure, limiting the effectiveness of CD8+ T cells through multiple mechanisms. These include the release of inhibitory cytokines (e.g., TGF-β, IL-10), the expression of IC molecules (e.g., PD-L1), and the accumulation of suppressive immune cell populations such as Tregs and MDSCs. Although CD8+ T cells possess potent cytotoxic mechanisms, only a fraction of tumor-infiltrating cells are fully functional, with many exhibiting exhaustions marked by elevated PD-1, TIM-3, or LAG-3 expression. Activation occurs through antigen presentation by DCs via the TCR and co-stimulatory molecules (e.g., CD28, CD27), which leads to the release of perforin, granzymes, and Fas ligand, as well as cytokines such as IFN-γ and TNF-α. However, the TME’s metabolic constraints - such as glutamine deprivation - can further impair mitochondrial function and induce apoptosis in CD8+ T cells, weakening their antitumor activity^[102]^. Recent studies have identified specific CD8+ T cell subpopulations associated with better prognosis in HCC^[103]^. High intratumoral levels of CD103+ CD8+ T cells, as well as PD-1+ CD161+ subsets, have been linked to improved survival^[104]^. Moreover, CXCR5+ CD8+ T cells have shown the ability to promote antitumor immunity by stimulating IL-21–mediated B cell activation and IgG production^[105]^. These findings underscore the clinical relevance of restoring CD8+ T cell function through ICIs and combination therapies aimed at overcoming the immunosuppressive features of the TME^[106]^.

However, tumors are categorized as cold and hot tumors based on their immune environment. Cold tumors have low T cell infiltration, high CAF and MDSC populations, immunosuppressive cytokines, and a lack of PD-L1 expression, making them unresponsive to immunotherapy^[107]^. Hot tumors show increased T cell infiltration, pro-inflammatory cytokines, PD-L1 expression, and CD8+ T cells, making them more susceptible to ICIs. Understanding these TME components opens avenues for developing effective immunotherapies targeting immune suppression and enhancing antitumor immunity^[108]^. In HCC, persistent chronic inflammation often leads to the upregulation of IC molecules, such as PD-1, PD-L1, and CTLA-4. This increased expression contributes to the functional exhaustion and apoptosis of CD8+ T cells, thereby impairing their antitumor efficacy. An effective therapeutic strategy to overcome this immune evasion involves blocking IC pathways that normally serve to regulate immune responses and maintain self-tolerance by limiting T cell activation. However, cancer cells often exploit them to escape immune-mediated destruction. Checkpoint inhibitors - including PD-1 blockers (e.g., nivolumab, pembrolizumab), PD-L1 inhibitors (e.g., atezolizumab, durvalumab), and CTLA-4 antagonists (e.g., tremelimumab, ipilimumab) - have shown promise in restoring CD8+ T cell function and antitumor immunity^[109]^. Nivolumab and pembrolizumab, both anti-PD-1 monoclonal antibodies, received accelerated FDA approval as second-line monotherapies for advanced HCC based on durable responses from early-phase trials (CheckMate-040 and KEYNOTE-224)^[110,111]^. However, subsequent phase III studies (CheckMate-459 and KEYNOTE-240) showed mixed results, underscoring the need for improved patient selection strategies^[112,113]^. These ICIs act by reactivating exhausted CD8+ T cells within the TME, enhancing T cell infiltration and IFN-γ–mediated cytotoxicity. Preclinical models have shown that combining PD-1/PD-L1 blockade with anti-angiogenic agents (e.g., VEGF inhibitors) or immune modulators can further improve efficacy by promoting DC maturation and reducing immunosuppressive MDSCs.

In this context, Atezolizumab (a monoclonal anti-PD-1 antibody) combined with Bevacizumab (a monoclonal anti-VEGF antibody) has been approved as a first-line therapy for advanced HCC^[114]^. The IMbrave150 trial demonstrated that Atezolizumab/Bevacizumab resulted in a 12-month OS rate of 67.2% compared to 54.6% in the sorafenib (400 mg BID) group. Further analyses showed a median OS of 19.2 *vs*. 13.4 months and progression-free survival (PFS) of 6.9 *vs*. 4.8 months for Atezolizumab/Bevacizumab and sorafenib, respectively, in patients with unresectable HCC^[115]^. The benefits of this combination were consistent across patients with BCLC stages B and C, extrahepatic metastases, and portal vein invasion. A multicenter, real-world retrospective analysis confirmed that the Atezolizumab/Bevacizumab combination was well tolerated. There were no treatment-related deaths or new adverse events, achieving a median OS of 14.9 months and PFS of 6.8 months^[116]^.

Nivolumab (anti-PD-1) received FDA approval in 2017 as second-line monotherapy after sorafenib for advanced HCC based on the CheckMate 040 phase I/II trial^[111]^. Similarly, pembrolizumab (anti-PD-1) was approved in 2018 following the Keynote-224 phase II study, which enrolled 104 patients with advanced HCC previously treated with sorafenib^[110]^. The trial demonstrated an overall response rate of 17% per RECIST v1.1, with a median PFS of 4.9 months and a median OS of 12.9 months. However, subsequent Phase III trials - CheckMate-459 and KEYNOTE-240 - produced mixed outcomes, emphasizing the need for improved strategies to identify patients most likely to benefit from treatment^[112,113]^.

Tremelimumab, targeting CTLA-4, especially in patients with HCV-related HCC, has demonstrated immunologic and clinical activity by enhancing tumor-specific T cell activation and intratumoral CD8+ infiltration^[117]^. Most recently, the HIMALAYA trial evaluated a single priming dose of the anti-CTLA-4 inhibitor tremelimumab 300 mg daily combined with the anti-PD-L1 agent durvalumab 1,500 mg every 4 weeks in patients with unresectable HCC. The trial demonstrated a significant improvement in median OS by 2.5 months (16.4 *vs*. 13.8 months) compared to sorafenib (400 mg twice daily). Thus, these findings have positioned the combination of tremelimumab and durvalumab as a recommended first-line therapy for patients with unresectable HCC^[118]^.

Clinical trials investigating adjuvant and neoadjuvant immunotherapies in early- or intermediate-stage HCC have shown encouraging results. Moreover, pilot neoadjuvant studies explore using anti-PD-1 antibodies alone or combined with anti-CTLA-4 antibodies^[118]^. Neoadjuvant immunotherapies have shown promising results in early-stage HCC, with improvements in T cell infiltration and pathological complete response rates. However, these treatments still need to be included in clinical guidelines due to limitations in trial designs and the absence of validation studies.

Additionally, the CheckMate 040 randomized clinical trial evaluated the therapeutic potential and safety profile of combining ipilimumab and nivolumab in patients with advanced HCC previously treated with sorafenib, suggesting that dual checkpoint blockade may offer clinical benefit even after prior immunotherapy^[119]^. Further validation through Phase III trials is essential before incorporating these strategies into clinical practice, and in this context, TME plays a fundamental role.

In addition to the PD-1/PD-L1 axis, recent studies have highlighted the critical role of the LAG-3/FGL1 axis in contributing to T cell exhaustion and resistance to ICIs in HCC^[120]^. LAG-3, a co-inhibitory receptor expressed on dysfunctional CD8+ T cells, interacts with its high-affinity ligand fibrinogen-like protein 1 (FGL1), which is often upregulated in HCC. This interaction suppresses T cell activation and promotes immune escape. Preclinical models have demonstrated that blockade of LAG-3 or FGL1 restores T cell activity and enhances antitumor immunity, particularly when used in combination with anti-PD-1/PD-L1 therapies^[120,121]^. Novel ICI combinations targeting both PD-1/PD-L1 and LAG-3/FGL1 pathways are currently under investigation and have shown synergistic effects in enhancing T cell infiltration and function within the TME^[122]^. These findings support the inclusion of LAG-1/FGL1-targeted strategies as a promising avenue in immunotherapy for HCC^[123]^.

Tumor resistance to ICIs presents a significant challenge and is driven by various factors. One such factor is the mutational burden in HCC, where total somatic mutations influence immune cell regulation^[124]^. A study analyzing 134 HCC samples showed that the activation of the Wnt/β-catenin pathway leads to “immune exclusion”, characterized by poor infiltration of CD8+ T cells in the TME. Indeed, HCC variants with β-catenin pathway activation exhibit significantly reduced CD8-positive T cell infiltration and PD-L1 expression, suggesting diminished responsiveness to PD-1/PD-L1 antibody therapies. However, tumors with Wnt/β-catenin pathway activation showed progressive disease compared to those without such mutations, which demonstrated complete/partial response and stable disease. PFS was markedly shorter in cases with Wnt/β-catenin activation (2.0 *vs.* 7.4 months, *P* < 0.0001)^[125]^. Another study analyzed 34 patients with unresectable HCC treated with anti-PD-1 antibodies. β-catenin pathway activation was found in 41.2% of cases, and three positive prognostic factors were identified: lack of β-catenin activation, PD-L1-combined positive score (CPS) ≥ 1, and high CD8-positive cell infiltration. Patients with more positive prognostic factors had significantly improved PFS and OS^[126]^. These findings highlight β-catenin activation as a key factor in ICI resistance, with poor CD8-positive cell infiltration and low PD-L1 expression associated with reduced therapeutic efficacy. However, due to the limited sample sizes, further research is needed to validate these observations.

A recent study evaluated the molecular analyses of tumor samples from 358 HCC patients participating in the IMbrave150 phase 3 trial. Key findings show that pre-existing immunity, characterized by high levels of CD274 expression, a T-effector gene signature, and dense intratumoral CD8+ T cells, was linked to improved clinical outcomes with combination therapy. Conversely, reduced benefit was observed in patients with a high Treg to effector T cell ratio and elevated expression of oncofetal genes such as GPC3 and AFP. Enhanced effectiveness of the combination therapy compared to atezolizumab alone was associated with high VEGF receptor 2 (KDR) expression and signatures of Tregs and myeloid inflammation^[127]^. Thus, the study suggested that anti-VEGF enhances the activity of anti-PD-L1 by addressing angiogenesis, Treg expansion, and myeloid cell-driven inflammation.

In addition to VEGF and PD-L1 regulation, several intracellular signaling pathways are involved in shaping the TME and modulating immune resistance in HCC. Among these, the MAPK/ERK and STAT3 pathways play key roles in promoting tumor progression, immune evasion, and resistance to immunotherapy. The MAPK pathway, often activated by receptor tyrosine kinases, regulates cell proliferation, survival, and cytokine expression in tumor cells and immune cells. Hyperactivation of this pathway contributes to the immunosuppressive microenvironment by enhancing PD-L1 expression, thus impairing T cell cytotoxicity^[128]^.

Meanwhile, the STAT3 signaling pathway, activated by cytokines such as IL-L6 and growth factors like VEGF, is a central regulator of immune tolerance and tumor-promoting inflammation. STAT3 upregulates immunosuppressive mediators including PD-L1, IL-10, and TGF-Beta, while inhibiting DC maturation and T cell activation. Persistent STAT3 activation in TAMs and MDSCs reinforces immunosuppression in the TME, limiting the efficacy of ICIs. Therefore, targeting the MAPK and STAT3 pathways has emerged as a strategy to overcome resistance to immunotherapy in HCC^[129]^. Ongoing trials are evaluating inhibitors of these pathways in combination with ICIs to restore immune surveillance and improve patient outcomes.

Other factors involved in tumor resistance to ICIs are inactivating mutations in TP53. The p53, a tumor suppressor encoded by TP53, not only regulates cancer cell behavior but also influences the immune response. Its loss or mutation in cancer cells can promote immune evasion by altering myeloid and T cell activity. Additionally, p53 functions in immune cells, with effects that may either suppress or support tumor growth^[130]^. In this context, understanding these roles could guide therapies targeting the differing p53 statuses in tumors and normal tissues. Furthermore, T cell exhaustion caused by the interaction of lymphocyte-activation gene 3 (LAG-3) molecules with overexpressed FGL1, a liver-secreted protein and a key ligand for LAG-3, in TME has been linked to poor prognosis and resistance to anti-PD-1 therapy^[131]^. Targeting ICs such as PD-L1 or LAG-3 with ICIs in HCC can reverse this T cell exhaustion in the TME. Pro-inflammatory cytokines such as type I interferons (IFN-I) play a key role in promoting T cell activation within the TME and creating favorable conditions for immunotherapy^[132]^. However, recent research indicated that prolonged inflammatory cytokine signaling contributes to the severe exhaustion of CD8+ T cells, resulting in reduced efficacy of ICIs and worse outcomes for HCC patients^[35]^.

MDSCs contribute to immunotherapy resistance through various mechanisms, including the production of MMP9 and angiogenic factors, recruitment via CCL2, and regulation of IDO by CARD9. They secrete cytokines such as IL-6, IL-10, and IL-23, promoting cell survival and activating Tregs, which suppress immunity. Additionally, MDSCs release NO, ROS, and peroxynitrite, impairing T cell function and migration. MDSC-derived EVs enhance immunosuppression via PD-L1 and TGF-β secretion, while ARG1 depletes L-arginine, weakening T cell responses^[133]^. Even TAMs can suppress T cells through PD-L1/PD-1 interactions, inducing immunosuppressive factors (TGF-β, IDO), and creating a hypoxic, immunosuppressive microenvironment. TANs can support immunoresistance by releasing cytokines (e.g., VEGF, MMP9) and promoting tumor growth^[134]^. They can also form NETs, which shield tumor cells, inhibit T cell infiltration, and trap activated T cells, particularly in liver fibrosis-associated environments. These mechanisms contribute to immune exclusion and HCC progression^[42]^. CAFs can create immunosuppressive niches through ECM remodeling, TGF-β signaling, and collagen deposition, which act as physical barriers to immune cell infiltration^[135]^. They also modulate Tregs, further enhancing immune suppression and resistance to therapy^[136]^.

Recently, Zhu *et al.* identified a gene signature, the atezolizumab–bevacizumab response signature (ABRS), with genes (CXCR2P1, ICOS, TIMD4, CTLA4, PAX5, KLRC3, FCRL3, AIM2, GBP5, and CCL4) involved in the regulation of T cell activation (CTLA4 and ICOS) and innate immunity (KLRC3, FCRL3, and AIM2), and associated with PFS after atezolizumab–bevacizumab initiation^[127]^. Then, using an AI model to predict the ABRS directly from histological slides, researchers found that high ABRS-P values were significantly associated with improved PFS. The authors performed a comprehensive scRNA-seq analysis of TILs across 21 cancer types from 316 patients. This effort resulted in the construction of a high-resolution atlas including 397,810 high-quality T cells, which revealed distinct T cell composition patterns and multiple state-transition pathways involved in CD8+ T cell exhaustion. By applying advanced bioinformatics techniques - including batch effect correction, clustering algorithms, and trajectory inference - the study uncovered the heterogeneity and dynamic states of TILs. Spatial transcriptomics further showed that areas with high ABRS-P values had elevated immune effector activity^[35]^. These results highlight the TME’s role in resistance and suggest AI-based approaches as valuable tools for understanding and overcoming therapeutic resistance.

Combining immunotherapy with TKIs or locoregional treatments such as tumor ablation, TACE, or TARE has shown potential, as these therapies enhance antigen presentation following tumor cell destruction^[137]^. Subsequent immune effects, called “abscopal” effects, can be amplified with ICIs^[138]^. Tremelimumab has been shown to increase CD8+ T cell infiltration in HCC patients, suggesting its potential combination with partial ablation or TACE. Early findings from the PETAL study, which explored pembrolizumab after TACE, indicate good tolerability. Ongoing Phase III trials are investigating combinations of other ICIs with TACE^[139]^. Additionally, adding anti-angiogenic drugs to locoregional therapy and ICIs may improve efficacy, with several Phase III trials currently evaluating this approach.

A Chinese study demonstrated significantly improved OS, PFS, and objective response rates with TACE plus Atezolizumab/Bevacizumab compared to Atezolizumab/Bevacizumab alone. TACE reduces the tumor burden and immunosuppressive factors like Treg cells while inducing hypoxia, which upregulates VEGF expression and enhances the efficacy of ICIs^[140]^.

Studies have shown that combining ICIs like tremelimumab (anti-CTLA-4) with local ablation enhances CD8+ T cell activity in the tumor periphery, resulting in promising antitumor effects^[141]^.

However, the etiology of the underlying liver disease may determine a different efficacy of immunotherapy due to TME. HCC of viral origin tends to be more immunogenic, responding better to ICIs. A systematic review analyzing systemic therapies for HCC revealed that immunotherapies were more effective in HCC with viral etiology than non-viral causes, contrasting with the performance of TKIs or anti-VEGF therapies. Metabolic dysfunction-associated steatotic liver disease (MASLD)-related HCC accumulates exhausted CD8+PD-1+ T cells in the TME, and preclinical studies showed that anti-PD-1 treatment led to tumor progression rather than regression in such cases^[142]^.

## CONCLUSIONS

In conclusion, the intricate TME in HCC plays a pivotal role in shaping tumor progression, therapeutic resistance, and immune evasion. Advances in locoregional therapies like TACE and SIRT and in systemic immunotherapies have demonstrated the potential to modulate the TME and improve patient outcomes. However, variability in treatment responses, influenced by factors such as the etiology of liver disease and tumor heterogeneity, underscores the need for personalized approaches. Future research focusing on integrating predictive biomarkers, innovative combination strategies, and a deeper understanding of TME dynamics will be crucial to optimizing HCC management and advancing toward more effective and tailored therapies.

## DECLARATIONS

### Copyright

© The Author(s) 2025.

## References

[1] Rumgay H, Arnold M, Ferlay J (2022). Global burden of primary liver cancer in 2020 and predictions to 2040. J Hepatol.

[2] Kulik L, El-Serag HB (2019). Epidemiology and management of hepatocellular carcinoma. Gastroenterology.

[3] Sung H, Ferlay J, Siegel RL (2021). Global cancer statistics 2020: GLOBOCAN estimates of incidence and mortality worldwide for 36 cancers in 185 countries. CA Cancer J Clin.

[4] Llovet JM, Kelley RK, Villanueva A (2021). Hepatocellular carcinoma. Nat Rev Dis Primers.

[5] Singal AG, Lampertico P, Nahon P (2020). Epidemiology and surveillance for hepatocellular carcinoma: new trends. J Hepatol.

[6] Villanueva A (2019). Hepatocellular carcinoma. N Engl J Med.

[7] (2018). European Association for the Study of the Liver. EASL Clinical Practice Guidelines: management of hepatocellular carcinoma. J Hepatol.

[8] Xu L, Kim Y, Spolverato G, Gani F, Pawlik TM (2016). Racial disparities in treatment and survival of patients with hepatocellular carcinoma in the United States. Hepatobiliary Surg Nutr.

[9] Altekruse SF, Henley SJ, Cucinelli JE, McGlynn KA (2014). Changing hepatocellular carcinoma incidence and liver cancer mortality rates in the United States. Am J Gastroenterol.

[10] Koza A, Bhogal RH, Fotiadis N, Mavroeidis VK (2023). The role of ablative techniques in the management of hepatocellular carcinoma: indications and outcomes. Biomedicines.

[11] Reig M, Forner A, Rimola J (2022). BCLC strategy for prognosis prediction and treatment recommendation: the 2022 update. J Hepatol.

[12] Llovet JM, De Baere T, Kulik L (2021). Locoregional therapies in the era of molecular and immune treatments for hepatocellular carcinoma. Nat Rev Gastroenterol Hepatol.

[13] Llovet JM, Castet F, Heikenwalder M (2022). Immunotherapies for hepatocellular carcinoma. Nat Rev Clin Oncol.

[14] Liu Y, Xun Z, Ma K (2023). Identification of a tumour immune barrier in the HCC microenvironment that determines the efficacy of immunotherapy. J Hepatol.

[15] Galasso L, Cerrito L, Maccauro V (2024). Hepatocellular carcinoma and the multifaceted relationship with its microenvironment: attacking the hepatocellular carcinoma defensive fortress. Cancers.

[16] Ladd AD, Duarte S, Sahin I, Zarrinpar A (2024). Mechanisms of drug resistance in HCC. Hepatology.

[17] Hinshaw DC, Shevde LA (2019). The tumor microenvironment innately modulates cancer progression. Cancer Res.

[18] Chen C, Wang Z, Ding Y, Qin Y (2023). Tumor microenvironment-mediated immune evasion in hepatocellular carcinoma. Front Immunol.

[19] Robinson MW, Harmon C, O’Farrelly C (2016). Liver immunology and its role in inflammation and homeostasis. Cell Mol Immunol.

[20] Hanahan D, Coussens LM (2012). Accessories to the crime: functions of cells recruited to the tumor microenvironment. Cancer Cell.

[21] Volponi C, Gazzillo A, Bonavita E (2022). The tumor microenvironment of hepatocellular carcinoma: untying an intricate immunological network. Cancers.

[22] Williams MA, Tyznik AJ, Bevan MJ (2006). Interleukin-2 signals during priming are required for secondary expansion of CD8+ memory T cells. Nature.

[23] Hao X, Sun G, Zhang Y (2021). Targeting immune cells in the tumor microenvironment of HCC: new opportunities and challenges. Front Cell Dev Biol.

[24] Wang H, Liu F, Wu X (2024). Cancer-associated fibroblasts contributed to hepatocellular carcinoma recurrence and metastasis via CD36-mediated fatty-acid metabolic reprogramming. Exp Cell Res.

[25] Su L, Yue Y, Yan Y (2024). Extracellular vesicles in hepatocellular carcinoma: unraveling immunological mechanisms for enhanced diagnosis and overcoming drug resistance. Front Immunol.

[26] Gupta T, Jarpula NS (2024). Hepatocellular carcinoma immune microenvironment and check point inhibitors-current status. World J Hepatol.

[27] Mignini I, Piccirilli G, Galasso L (2024). From the colon to the liver: how gut microbiota may influence colorectal cancer metastatic potential. J Clin Med.

[28] Nishida N, Kudo M (2017). Immunological microenvironment of hepatocellular carcinoma and its clinical implication. Oncology.

[29] Bai Y, Chen D, Cheng C (2022). Immunosuppressive landscape in hepatocellular carcinoma revealed by single-cell sequencing. Front Immunol.

[30] Xia C, Chen Y, Zhu Y (2024). Identification of DLAT as a potential therapeutic target via a novel cuproptosis-related gene signature for the prediction of liver cancer prognosis. J Gastrointest Oncol.

[31] Cariani E, Missale G (2019). Immune landscape of hepatocellular carcinoma microenvironment: implications for prognosis and therapeutic applications. Liver Int.

[32] Barry KC, Hsu J, Broz ML (2018). A natural killer-dendritic cell axis defines checkpoint therapy-responsive tumor microenvironments. Nat Med.

[33] Böttcher JP, Bonavita E, Chakravarty P (2018). NK cells stimulate recruitment of cDC1 into the tumor microenvironment promoting cancer immune control. Cell.

[34] Chen DS, Mellman I (2013). Oncology meets immunology: the cancer-immunity cycle. Immunity.

[35] Zheng L, Qin S, Si W (2021). Pan-cancer single-cell landscape of tumor-infiltrating T cells. Science.

[36] Ma L, Hernandez MO, Zhao Y (2019). Tumor cell biodiversity drives microenvironmental reprogramming in liver cancer. Cancer Cell.

[37] Iñarrairaegui M, Melero I, Sangro B (2018). Immunotherapy of hepatocellular carcinoma: facts and hopes. Clin Cancer Res.

[38] Peng W, Chen JQ, Liu C (2016). Loss of PTEN promotes resistance to T cell-mediated immunotherapy. Cancer Discov.

[39] Lu C, Rong D, Zhang B (2019). Current perspectives on the immunosuppressive tumor microenvironment in hepatocellular carcinoma: challenges and opportunities. Mol Cancer.

[40] Jaillon S, Ponzetta A, Di Mitri D, Santoni A, Bonecchi R, Mantovani A (2020). Neutrophil diversity and plasticity in tumour progression and therapy. Nat Rev Cancer.

[41] Zhou SL, Zhou ZJ, Hu ZQ (2016). Tumor-associated neutrophils recruit macrophages and T-regulatory cells to promote progression of hepatocellular carcinoma and resistance to sorafenib. Gastroenterology.

[42] Yang LY, Luo Q, Lu L (2020). Increased neutrophil extracellular traps promote metastasis potential of hepatocellular carcinoma via provoking tumorous inflammatory response. J Hematol Oncol.

[43] Sun R, Xiong Y, Liu H (2020). Tumor-associated neutrophils suppress antitumor immunity of NK cells through the PD-L1/PD-1 axis. Transl Oncol.

[44] Guan X, Lu Y, Zhu H (2021). The crosstalk between cancer cells and neutrophils enhances hepatocellular carcinoma metastasis via neutrophil extracellular traps-associated cathepsin G component: a potential therapeutic target. J Hepatocell Carcinoma.

[45] Li XF, Chen DP, Ouyang FZ (2015). Increased autophagy sustains the survival and pro-tumourigenic effects of neutrophils in human hepatocellular carcinoma. J Hepatol.

[46] Thommen DS, Koelzer VH, Herzig P (2018). A transcriptionally and functionally distinct PD-1^+^ CD8^+^ T cell pool with predictive potential in non-small-cell lung cancer treated with PD-1 blockade. Nat Med.

[47] Kunitani H, Shimizu Y, Murata H, Higuchi K, Watanabe A (2002). Phenotypic analysis of circulating and intrahepatic dendritic cell subsets in patients with chronic liver diseases. J Hepatol.

[48] Thaiss CA, Semmling V, Franken L, Wagner H, Kurts C (2011). Chemokines: a new dendritic cell signal for T cell activation. Front Immunol.

[49] Chen C, Ma YH, Zhang YT (2018). Effect of dendritic cell-based immunotherapy on hepatocellular carcinoma: a systematic review and meta-analysis. Cytotherapy.

[50] Huang Y, Ge W, Zhou J, Gao B, Qian X, Wang W (2021). The role of tumor associated macrophages in hepatocellular carcinoma. J Cancer.

[51] Dong P, Ma L, Liu L (2016). CD86^+^/CD206^+^, diametrically polarized tumor-associated macrophages, predict hepatocellular carcinoma patient prognosis. Int J Mol Sci.

[52] Zhu Y, Yang J, Xu D (2019). Disruption of tumour-associated macrophage trafficking by the osteopontin-induced colony-stimulating factor-1 signalling sensitises hepatocellular carcinoma to anti-PD-L1 blockade. Gut.

[53] Zhu XD, Zhang JB, Zhuang PY (2008). High expression of macrophage colony-stimulating factor in peritumoral liver tissue is associated with poor survival after curative resection of hepatocellular carcinoma. J Clin Oncol.

[54] Veglia F, Sanseviero E, Gabrilovich DI (2021). Myeloid-derived suppressor cells in the era of increasing myeloid cell diversity. Nat Rev Immunol.

[55] Hoechst B, Voigtlaender T, Ormandy L (2009). Myeloid derived suppressor cells inhibit natural killer cells in patients with hepatocellular carcinoma via the NKp30 receptor. Hepatology.

[56] Erez N, Truitt M, Olson P, Arron ST, Hanahan D (2010). Cancer-associated fibroblasts are activated in incipient neoplasia to orchestrate tumor-promoting inflammation in an NF-kappaB-dependent manner. Cancer Cell.

[57] Deng Y, Cheng J, Fu B (2017). Hepatic carcinoma-associated fibroblasts enhance immune suppression by facilitating the generation of myeloid-derived suppressor cells. Oncogene.

[58] Zhang Q, He Y, Luo N (2019). Landscape and dynamics of single immune cells in hepatocellular carcinoma. Cell.

[59] Guo X, Zhang Y, Zheng L (2018). Global characterization of T cells in non-small-cell lung cancer by single-cell sequencing. Nat Med.

[60] Newell EW, Cheng Y (2016). Mass cytometry: blessed with the curse of dimensionality. Nat Immunol.

[61] Giesen C, Wang HA, Schapiro D (2014). Highly multiplexed imaging of tumor tissues with subcellular resolution by mass cytometry. Nat Methods.

[62] Wan JCM, Massie C, Garcia-Corbacho J (2017). Liquid biopsies come of age: towards implementation of circulating tumour DNA. Nat Rev Cancer.

[63] (2025). European Association for the Study of the Liver. EASL Clinical Practice Guidelines on the management of hepatocellular carcinoma. J Hepatol.

[64] Forner A, Gilabert M, Bruix J, Raoul JL (2014). Treatment of intermediate-stage hepatocellular carcinoma. Nat Rev Clin Oncol.

[65] (2021). Ebeling Barbier C, Heindryckx F, Lennernäs H. Limitations and possibilities of transarterial chemotherapeutic treatment of hepatocellular carcinoma. Int J Mol Sci.

[66] Liu Z, Tu K, Wang Y (2017). Hypoxia accelerates aggressiveness of hepatocellular carcinoma cells involving oxidative stress, epithelial-mesenchymal transition and non-canonical hedgehog signaling. Cell Physiol Biochem.

[67] (2016). de Baere T, Plotkin S, Yu R, Sutter A, Wu Y, Cruise GM. An in vitro evaluation of four types of drug-eluting microspheres loaded with doxorubicin. J Vasc Interv Radiol.

[68] Kim MJ, Jang JW, Oh BS (2013). Change in inflammatory cytokine profiles after transarterial chemotherapy in patients with hepatocellular carcinoma. Cytokine.

[69] Padia SA (2019). Is idarubicin the future of TACE?. Radiology.

[70] Katayama K, Imai T, Abe Y (2018). Number of nodules but not size of hepatocellular carcinoma can predict refractoriness to transarterial chemoembolization and poor prognosis. J Clin Med Res.

[72] Choi J, Lee D, Shim JH (2020). Evaluation of transarterial chemoembolization refractoriness in patients with hepatocellular carcinoma. PLoS One.

[73] Tak E, Lee S, Lee J (2011). Human carbonyl reductase 1 upregulated by hypoxia renders resistance to apoptosis in hepatocellular carcinoma cells. J Hepatol.

[74] Singh P, Toom S, Avula A, Kumar V, Rahma OE (2020). The immune modulation effect of locoregional therapies and its potential synergy with immunotherapy in hepatocellular carcinoma. J Hepatocell Carcinoma.

[75] Tampaki M, Ionas E, Hadziyannis E, Deutsch M, Malagari K, Koskinas J (2020). Association of TIM-3 with BCLC stage, serum PD-L1 detection, and response to transarterial chemoembolization in patients with hepatocellular carcinoma. Cancers.

[76] Jekarl DW, Lee S, Kwon JH (2019). Complex interaction networks of cytokines after transarterial chemotherapy in patients with hepatocellular carcinoma. PLoS One.

[77] Pinato DJ, Karamanakos G, Arizumi T (2014). Dynamic changes of the inflammation-based index predict mortality following chemoembolisation for hepatocellular carcinoma: a prospective study. Aliment Pharmacol Ther.

[78] Pinato DJ, Murray SM, Forner A (2021). Trans-arterial chemoembolization as a loco-regional inducer of immunogenic cell death in hepatocellular carcinoma: implications for immunotherapy. J Immunother Cancer.

[79] Tan J, Fan W, Liu T (2023). TREM2^+^ macrophages suppress CD8^+^ T-cell infiltration after transarterial chemoembolisation in hepatocellular carcinoma. J Hepatol.

[80] Cappelli A, Cucchetti A, Cabibbo G (2016). Refining prognosis after trans-arterial chemo-embolization for hepatocellular carcinoma. Liver Int.

[81] Kadalayil L, Benini R, Pallan L (2013). A simple prognostic scoring system for patients receiving transarterial embolisation for hepatocellular cancer. Ann Oncol.

[82] Tang Y, Wu Y, Xue M, Zhu B, Fan W, Li J (2022). A 10-gene signature identified by machine learning for predicting the response to transarterial chemoembolization in patients with hepatocellular carcinoma. J Oncol.

[83] Fako V, Martin SP, Pomyen Y (2019). Gene signature predictive of hepatocellular carcinoma patient response to transarterial chemoembolization. Int J Biol Sci.

[84] Huang M, Wang L, Chen J (2016). Regulation of COX-2 expression and epithelial-to-mesenchymal transition by hypoxia-inducible factor-1α is associated with poor prognosis in hepatocellular carcinoma patients post TACE surgery. Int J Oncol.

[85] He Q, Yang J, Jin Y (2022). Development and validation of TACE refractoriness-related diagnostic and prognostic scores and characterization of tumor microenvironment infiltration in hepatocellular carcinoma. Front Immunol.

[86] Wang L, Cao J, Liu Z (2024). Enhanced interactions within microenvironment accelerates dismal prognosis in HBV-related HCC after TACE. Hepatol Commun.

[87] Yue Y, Ren Z, Wang Y (2024). Impact of microparticle transarterial chemoembolization (mTACE) on myeloid-derived suppressor cell subtypes in hepatocellular carcinoma: clinical correlations and therapeutic implications. Immun Inflamm Dis.

[88] Wang C, Leng B, You R (2024). A transcriptomic biomarker for predicting the response to TACE correlates with the tumor microenvironment and radiomics features in hepatocellular carcinoma. J Hepatocell Carcinoma.

[89] Ayaru L, Pereira SP, Alisa A (2007). Unmasking of alpha-fetoprotein-specific CD4^+^ T cell responses in hepatocellular carcinoma patients undergoing embolization. J Immunol.

[90] Xue J, Ni H, Wang F, Xu K, Niu M (2021). Advances in locoregional therapy for hepatocellular carcinoma combined with immunotherapy and targeted therapy. J Interv Med.

[91] El Fouly A, Ertle J, El Dorry A (2015). In intermediate stage hepatocellular carcinoma: radioembolization with yttrium 90 or chemoembolization?. Liver Int.

[92] Kallini JR, Gabr A, Salem R, Lewandowski RJ (2016). Transarterial radioembolization with Yttrium-90 for the treatment of hepatocellular carcinoma. Adv Ther.

[93] Salem R, Lewandowski RJ, Mulcahy MF (2010). Radioembolization for hepatocellular carcinoma using Yttrium-90 microspheres: a comprehensive report of long-term outcomes. Gastroenterology.

[94] Franzè MS, Vigneron P, Sessa A (2025). Prognostic factors influencing outcomes in hepatocellular carcinoma patients undergoing selective internal radiation therapy. Ann Hepatol.

[95] Lugade AA, Sorensen EW, Gerber SA, Moran JP, Frelinger JG, Lord EM (2008). Radiation-induced IFN-gamma production within the tumor microenvironment influences antitumor immunity. J Immunol.

[96] Barcellos-Hoff MH, Park C, Wright EG (2005). Radiation and the microenvironment - tumorigenesis and therapy. Nat Rev Cancer.

[97] Chew V, Tow C, Teo M (2010). Inflammatory tumour microenvironment is associated with superior survival in hepatocellular carcinoma patients. J Hepatol.

[98] Chew V, Lee YH, Pan L (2019). Immune activation underlies a sustained clinical response to Yttrium-90 radioembolisation in hepatocellular carcinoma. Gut.

[99] Bitar R, Salem R, Finn R, Greten TF, Goldberg SN, Chapiro J (2024). Interventional oncology meets immuno-oncology: combination therapies for hepatocellular carcinoma. Radiology.

[100] Giraud J, Chalopin D, Blanc JF, Saleh M (2021). Hepatocellular carcinoma immune landscape and the potential of immunotherapies. Front Immunol.

[101] Farhood B, Najafi M, Mortezaee K (2019). CD8^+^ cytotoxic T lymphocytes in cancer immunotherapy: a review. J Cell Physiol.

[102] Wherry EJ, Kurachi M (2015). Molecular and cellular insights into T cell exhaustion. Nat Rev Immunol.

[103] Chen L, Huang H, Huang Z (2023). Prognostic values of tissue-resident CD8^+^T cells in human hepatocellular carcinoma and intrahepatic cholangiocarcinoma. World J Surg Oncol.

[104] Li Z, Zheng B, Qiu X (2020). The identification and functional analysis of CD8+PD-1+CD161+ T cells in hepatocellular carcinoma. NPJ Precis Oncol.

[105] Ye L, Li Y, Tang H (2019). CD8+CXCR5+T cells infiltrating hepatocellular carcinomas are activated and predictive of a better prognosis. Aging.

[106] Abushukair HM, Saeed A (2022). Hepatocellular carcinoma and immunotherapy: beyond immune checkpoint inhibitors. World J Gastrointest Oncol.

[107] Duan Q, Zhang H, Zheng J, Zhang L (2020). Turning cold into hot: firing up the tumor microenvironment. Trends Cancer.

[108] Ailia MJ, Heo J, Yoo SY (2023). Navigating through the PD-1/PDL-1 landscape: a systematic review and meta-analysis of clinical outcomes in hepatocellular carcinoma and their influence on immunotherapy and tumor microenvironment. Int J Mol Sci.

[109] Kudo M (2019). Pembrolizumab for the treatment of hepatocellular carcinoma. Liver Cancer.

[111] El-Khoueiry AB, Sangro B, Yau T (2017). Nivolumab in patients with advanced hepatocellular carcinoma (CheckMate 040): an open-label, non-comparative, phase 1/2 dose escalation and expansion trial. Lancet.

[113] Sangro B, Park J, Finn R (2020). LBA-3 CheckMate 459: long-term (minimum follow-up 33.6 months) survival outcomes with nivolumab versus sorafenib as first-line treatment in patients with advanced hepatocellular carcinoma. Ann Oncol.

[115] Salem R, Li D, Sommer N (2021). Characterization of response to atezolizumab+bevacizumab versus sorafenib for hepatocellular carcinoma: results from the IMbrave150 trial. Cancer Med.

[116] D’Alessio A, Fulgenzi CAM, Nishida N (2022). Preliminary evidence of safety and tolerability of atezolizumab plus bevacizumab in patients with hepatocellular carcinoma and Child-Pugh A and B cirrhosis: a real-world study. Hepatology.

[117] Sangro B, Gomez-Martin C, de la Mata M (2013). A clinical trial of CTLA-4 blockade with tremelimumab in patients with hepatocellular carcinoma and chronic hepatitis C. J Hepatol.

[118] Abou-alfa GK, Chan SL, Kudo M (2022). Phase 3 randomized, open-label, multicenter study of tremelimumab (T) and durvalumab (D) as first-line therapy in patients (pts) with unresectable hepatocellular carcinoma (uHCC): HIMALAYA. J Clin Oncol.

[119] Yau T, Kang YK, Kim TY (2020). Efficacy and safety of nivolumab plus ipilimumab in patients with advanced hepatocellular carcinoma previously treated with sorafenib: the CheckMate 040 randomized clinical trial. JAMA Oncol.

[120] Zuo D, Zhu Y, Wang K (2024). A novel LAG3 neutralizing antibody improves cancer immunotherapy by dual inhibition of MHC-II and FGL1 ligand binding. Biomed Pharmacother.

[121] Yang C, Qian Q, Zhao Y (2023). Fibrinogen-like protein 1 promotes liver-resident memory T-cell exhaustion in hepatocellular carcinoma. Front Immunol.

[122] Hua N, Chen A, Yang C (2022). The correlation of fibrinogen-like protein-1 expression with the progression and prognosis of hepatocellular carcinoma. Mol Biol Rep.

[123] Guo M, Yuan F, Qi F (2020). Expression and clinical significance of LAG-3, FGL1, PD-L1 and CD8^+^T cells in hepatocellular carcinoma using multiplex quantitative analysis. J Transl Med.

[124] Morita M, Nishida N, Aoki T (2023). Role of β-catenin activation in the tumor immune microenvironment and immunotherapy of hepatocellular carcinoma. Cancers.

[125] Harding JJ, Nandakumar S, Armenia J (2019). Prospective genotyping of hepatocellular carcinoma: clinical implications of next-generation sequencing for matching patients to targeted and immune therapies. Clin Cancer Res.

[126] Morita M, Nishida N, Sakai K (2021). Immunological microenvironment predicts the survival of the patients with hepatocellular carcinoma treated with anti-PD-1 antibody. Liver Cancer.

[127] Zhu AX, Abbas AR, de Galarreta MR (2022). Molecular correlates of clinical response and resistance to atezolizumab in combination with bevacizumab in advanced hepatocellular carcinoma. Nat Med.

[128] Zhang C, Hu S, Yin C, Wang G, Liu P (2025). STAT3 orchestrates immune dynamics in hepatocellular carcinoma: a pivotal nexus in tumor progression. Crit Rev Oncol Hematol.

[129] Wang Y, Wang W, Liu K (2024). The mechanism of Xihuang pills’ intervention in the tumour immune microenvironment for the treatment of liver cancer based on the STAT3-PDL1 pathway. J Ethnopharmacol.

[130] Blagih J, Buck MD, Vousden KH (2020). p53, cancer and the immune response. J Cell Sci.

[131] Wang J, Sanmamed MF, Datar I (2019). Fibrinogen-like protein 1 is a major immune inhibitory ligand of LAG-3. Cell.

[132] Yu R, Zhu B, Chen D (2022). Type I interferon-mediated tumor immunity and its role in immunotherapy. Cell Mol Life Sci.

[133] Fu Y, Guo X, Sun L (2024). Exploring the role of the immune microenvironment in hepatocellular carcinoma: implications for immunotherapy and drug resistance. Elife.

[134] Kuang DM, Zhao Q, Wu Y (2011). Peritumoral neutrophils link inflammatory response to disease progression by fostering angiogenesis in hepatocellular carcinoma. J Hepatol.

[135] Costa A, Kieffer Y, Scholer-Dahirel A (2018). Fibroblast heterogeneity and immunosuppressive environment in human breast cancer. Cancer Cell.

[136] Mariathasan S, Turley SJ, Nickles D (2018). TGFβ attenuates tumour response to PD-L1 blockade by contributing to exclusion of T cells. Nature.

[137] Gudd CLC, Au L, Triantafyllou E (2021). Activation and transcriptional profile of monocytes and CD8^+^ T cells are altered in checkpoint inhibitor-related hepatitis. J Hepatol.

[138] Pinato DJ, Kaseb A, Wang Y (2020). Impact of corticosteroid therapy on the outcomes of hepatocellular carcinoma treated with immune checkpoint inhibitor therapy. J Immunother Cancer.

[139] Kelley RK, Sangro B, Harris W (2021). Safety, efficacy, and pharmacodynamics of tremelimumab plus durvalumab for patients with unresectable hepatocellular carcinoma: randomized expansion of a phase I/II study. J Clin Oncol.

[140] Cao F, Shi C, Zhang G, Luo J, Zheng J, Hao W (2023). Improved clinical outcomes in advanced hepatocellular carcinoma treated with transarterial chemoembolization plus atezolizumab and bevacizumab: a bicentric retrospective study. BMC Cancer.

[141] Cui TM, Liu Y, Wang JB, Liu LX (2020). Adverse effects of immune-checkpoint inhibitors in hepatocellular carcinoma. Onco Targets Ther.

[142] Cheu JW, Wong CC (2024). The immune microenvironment of steatotic hepatocellular carcinoma: current findings and future prospects. Hepatol Commun.

